# An inducible germ cell ablation chicken model for high-grade germline chimeras

**DOI:** 10.1242/dev.202079

**Published:** 2023-09-25

**Authors:** Yi-Chen Chen, Daisuke Saito, Takayuki Suzuki, Tatsuya Takemoto

**Affiliations:** ^1^ Division of Research and Development, Setsuro Tech Inc., Tokushima 770-8503, Japan; ^2^ Laboratory for Embryology, Institute for Advanced Medical Sciences, Tokushima University, Tokushima 770-8503, Japan; ^3^ Department of Biology, Faculty of Science, Kyushu University, Fukuoka 819-0395, Japan; ^4^ Department of Biology, Graduate School of Science, Osaka Metropolitan University, Osaka 558-8585, Japan

**Keywords:** Chicken primordial germ cell, MAD7 nuclease, Nitroreductase/metronidazole, Cell ablation

## Abstract

Chicken embryos are a powerful and widely used animal model in developmental biology studies. Since the development of CRISPR technology, gene-edited chickens have been generated by transferring primordial germ cells (PGCs) into recipients after genetic modifications. However, low inheritance caused by competition between host germ cells and the transferred cells is a common complication and greatly reduces production efficiency. Here, we generated a gene-edited chicken, in which germ cells can be ablated in a drug-dependent manner, as recipients for gene-edited PGC transfer. We used the nitroreductase/metronidazole (NTR/Mtz) system for cell ablation, in which nitroreductase produces cytotoxic alkylating agents from administered metronidazole, causing cell apoptosis. The chicken Vasa homolog (*CVH*) gene locus was used to drive the expression of the nitroreductase gene in a germ cell-specific manner. In addition, a fluorescent protein gene, *mCherry*, was also placed in the *CVH* locus to visualize the PGCs. We named this system ‘germ cell-specific autonomous removal induction’ (gSAMURAI). gSAMURAI chickens will be an ideal recipient to produce offspring derived from transplanted exogenous germ cells.

## Teaser

**Summary:** A chicken model, termed gSAMURAI, has been developed that can be used as the surrogate for PGC-mediated gene editing, producing a high-grade germline chimera by inducible ablation of host germ cells.

## INTRODUCTION

Chickens, as an oviparous organism, exhibit an almost comprehensive embryonic development process *in ovo* and comparatively accessible incubation conditions compared with other animals, making them an ideal model for research in embryology that has been used since the times of ancient Greece (Zagris, 2022). For example, the accessibility of the chicken model facilitates embryo manipulation for the generation of quail–chick chimeras by xenogeneic tissue grafting, thus contributing to deciphering the ontogeny of the neural crest and of the immune system (Le Douarin, 1988). The use of an *ex ovo* embryo culture system (‘New's culture’) and embryo electroporation, together with advances in research techniques for studying gene regulation and function in development, have led to chickens becoming widely used non-mammalian model animals (New, 1955; Uchikawa et al., 2004). Although chicken embryos provide convenience for developmental biologists, the difficulty of transgenic chicken production has limited the merits of chicken utilization in developmental biology studies. Therefore, the availability of a simple method for the generation of transgenic chickens would expand the usefulness of chickens and lead to revolutions in understanding developmental regulation.

The production of transgenic chickens has proved to be difficult, and their unique reproductive system presents obstacles to researchers. In mammalian models, the one-cell-stage zygote is accessible and can be utilized to generate transgenic animals. However, eggs shed from the chicken oviduct have already developed to a high cell number; the one-cell-stage zygote of the chicken emerges in the infundibulum of the reproductive tract and fertilization is polyspermy, making production of transgenic chickens problematic. Therefore, transgenesis in chickens has mainly depended on the viral transfection system (Sang, 1994). Another strategy to generate transgenic animals is to utilize embryonic stem cells. Although chicken embryonic stem cells (cESCs) have been established, contributions to germline lineage and offspring production from cESCs have not been reported (Lavial and Pain, 2010; Pain et al., 1996; van de Lavoir et al., 2006b). Therefore, there is a huge difference in the availability of gene-edited organisms between chickens and other species, particularly since the development of CRISPR technology.

Nevertheless, by using genetic modification in the germline, genetically modified chicken offspring can be obtained (Salter et al., 1987). For better efficiency of germline transmission, scientists have focused on the improvement of *in vitro* culture methods and genetic manipulations of chicken primordial germ cells (PGCs) (van de Lavoir et al., 2006a). With many of their contributions, this PGC-mediated gene-editing technique has become widely used, not only in basic scientific research to identify the mechanism of germ cell development or sexual determination, but also for industrial purposes, such as the generation of transgenic chickens for recombinant protein production and breeding of low-allergen chickens (Ching et al., 2018; Choi et al., 2022; Ioannidis et al., 2021; Oishi et al., 2016).

However, the major disadvantage of this method is the low germline transmission efficiency caused by the competition between recipient host germ cells and donor cells. In addition, it is known that the efficiency becomes lower when chicken PGCs undergo long-term *in vitro* culture or manipulation (Collarini et al., 2015; Woodcock et al., 2019). To solve this problem, chemical or irradiation methods have been adopted to remove the endogenous germ cells of the recipient embryo before PGC transfer, with higher mortality in the treated embryo found to be a major side effect (Nakamura et al., 2012, 2010). Trefil et al. (2017) irradiated the adult testis, not the embryo, to avoid high mortality, but it took 2 weeks or more for the irradiation and 5 months for the restoration of spermatogenesis after transplantation (Koslova et al., 2020). Another group utilized an inter-species hybrid of guinea fowl and domestic fowl (chicken) as surrogates. They confirmed the sterility of the hybrid, but did not demonstrate offspring production (Molnar et al., 2019). Recently, genetic modification methods were also applied to generate surrogates for PGC transplantation. Taylor et al. (2017) developed a genetically sterile model by knocking out chicken Vasa homolog [*CVH*; also known as DEAD-box helicase 4 (*DDX4*)], which resulted in perfect germline transmission from donor cells. However, the frequency of obtaining sterile recipients was only 25% and restricted to females because of the limited intercrosses: the *CVH* heterozygous knockout rooster (CVH^KO/Z^) and the wild-type (WT) hens (CVH^Z/W^). They also produced a model carrying a germ cell-specific inducible caspase-9 (DAZL-iCaspase9) (Ballantyne et al., 2021). They obtained chicken offspring from donor PGCs with high efficiency by the induction of DAZL-iCaspase9 to ablate host germ cells, indicating that an inducible sterile model is suitable for surrogate purposes.

In this study, we have established a new chicken model to be used as a recipient chicken for PGC transplantation, in which host germ cells can be removed in a drug-dependent manner. We utilized the nitroreductase/metronidazole (NTR/Mtz) system for cell ablation, in which NTR produces cytotoxic alkylating agents from administered Mtz, causing cell apoptosis (Chen et al., 2011; Curado et al., 2008).

## RESULTS

### Design of the germ cell-specific autonomous removal induction (gSAMURAI) chicken

**Fig. 1.**
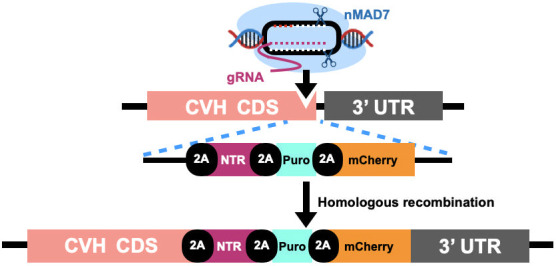
**Schematic of gSAMURAI chicken.** The bacterial nitroreductase (*NTR*) gene, puromycin-resistant gene (*Puro*) and *mCherry* gene are inserted into the 3’ end of the chicken Vasa homolog (*CVH*) coding sequence via the 2A peptide sequence (2A) using gene editing-mediated homologous recombination.

To remove endogenous germ cells in a drug-dependent manner, we applied the NTR/Mtz system (Curado et al., 2008). We inserted a bacterial nitroreductase (*NTR*) gene into the 3′ end of the chicken Vasa homolog (*CVH*) coding sequence via the 2A peptide sequence using gene editing-mediated homologous recombination. A puromycin-resistant (puro) gene for the selection of recombinants in PGCs and the mCherry gene for the visualization of PGCs were also inserted into the same locus (Fig. 1).

### Selection of crRNA of MAD7 nuclease for optimized gene editing efficiency

**Fig. 2.**
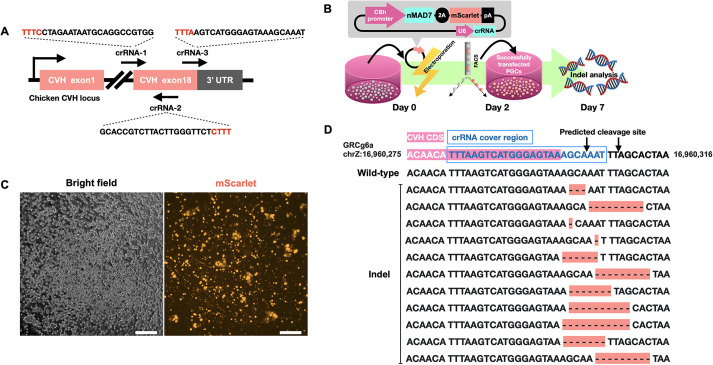
**Evaluation of MAD7 crRNAs showing high indel formation in chicken PGCs.** (A) Three MAD7 crRNAs targeting the C terminus of the *CVH* coding region were designed. The recognition sequence of each MAD7 crRNA is shown, and each PAM site is labeled in red. (B) Experimental procedure of plasmid transfection and the analysis of indel formation. First, the plasmid expressing nMAD7, crRNA and mScarlet was electroporated into PGCs. Second, successfully transfected cells with positive mScarlet expression were harvested by FACS at day 2 post-transfection. Finally, at day 7 post-transfection, all sorted cells were subjected to DNA extraction for further indel analysis by NGS. (C) mScarlet could be detected in PGCs 48 h after electroporation. Scale bars: 50 µm. Images are representative of more than three samples. (D) NGS analysis data of PGCs electroporated with crRNA-3. Multinucleotide deletions (highlighted) around the predicted cleavage site (arrows) were found.

The higher the double-stranded break (DSB) efficiency, the higher the knock-in (KI) efficiency. To this end, we first identified the optimized crRNA to introduce mutations into the *CVH* gene using chicken PGCs. Three MAD7 CRISPR RNAs (crRNAs) targeting the 3′ end of the *CVH* gene were designed, and their insertion-deletion mutation (indel) rates, which reflect DSB efficiency, were examined (Fig. 2A). We constructed a plasmid expressing each crRNA, MAD7 nuclease plus a C-terminal nuclear localization signal (NLS) (nMAD7), and mScarlet ubiquitously using U6 or CBh promoters. Each plasmid was introduced into chicken PGCs by electroporation. PGCs were derived from blood vessels of embryonic day 3 (E3) Barred Plymouth Rock (BPR) strain embryos, undergoing approximately 3 weeks of *in vitro* propagation until the cell number was sufficient for further studies. At day 2 post-transfection, successfully transfected cells were collected by fluorescence-activated cell sorting (FACS) using positive expression of mScarlet (Fig. 2B,C), and genomic DNA was extracted for indel analysis at each target site. Sequence analysis of genomic PCR amplicons revealed that among these three crRNAs number 3 had the highest indel frequency at 9.59% (indel read/total read: 105/1059), compared with number 1 and number 2 crRNAs, which presented 1.99% (indel read/total read: 20/1003) and 1.32% (indel read/total read: 14/1064), respectively (Table 1). We used crRNA number 3 (Fig. 2D) for further experiments.

**Table 1.**
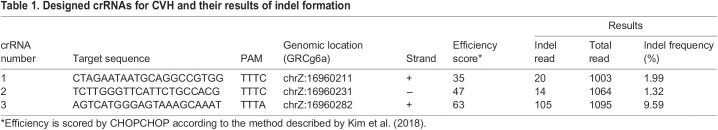
Designed crRNAs for CVH and their results of indel formation

### Establishment of PGCs carrying the gSAMURAI system by DNA cleavage-mediated homologous recombination

**Fig. 3.**
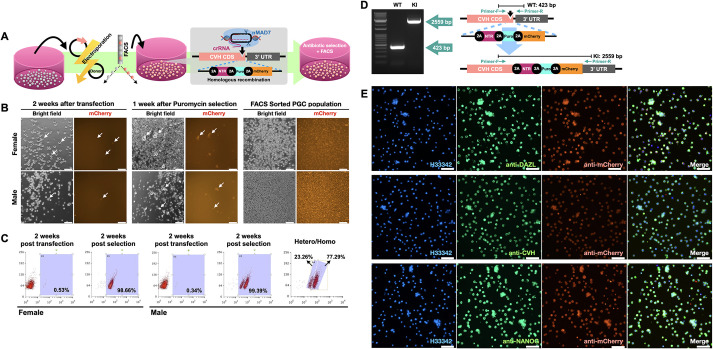
**Generation of gSAMURAI PGCs utilizing DNA cleavage-mediated homologous recombination.** (A) Illustration of the flowchart for the establishment of genetically modified PGCs through DNA cleavage-mediated homologous recombination. (B) The derivation and enrichment of genetically modified PGCs after transfection, antibiotic selection and FACS. (C) Cell population gating by the mCherry fluorescence intensity in PGCs after transfection and antibiotic selection. (D) PCR amplification using Primer-F and Primer-R for transgene insertion in *CVH* loci and the DNA sequences of amplicons. (E) Immunostaining of PGC markers and mCherry in gSAMURAI PGCs. H33342, Hoechst 33342 (nuclear stain). Scale bars: 50 µm. Images are representative of more than three samples.

Using the MAD7 expression plasmid and the donor plasmid, homologous recombinant PGCs were selected by mCherry fluorescence and puromycin resistance (Fig. 3A). As illustrated in Fig. 3B, mCherry-expressing PGCs were found in cell populations of both sexes in the second week after transfection at 0.53% (female) and 0.34% (male). After puromycin exposure for 1 week, the mCherry-expressing PGC rate was obviously enriched. Using a FACS sorter for quantification, 98.66% (female) and 99.39% (male) of mCherry-positive PGCs were detected after 2 weeks of antibiotic selection (Fig. 3B,C), indicating that the puromycin resistance gene supported the cell enrichment of KI PGCs. Moreover, two populations with different mCherry fluorescent intensities were found in the male PGCs, suggesting heterozygous and homozygous transgene insertion in *CVH* (Fig. 3C). To confirm successful knock-in, we performed genomic PCR analysis, followed by sequence analysis, using genomic DNA samples extracted from WT PGCs and PGCs with high mCherry intensity (Fig. 3D). The size and DNA sequences of each amplicon demonstrated that the transgene had been inserted into the targeted loci as in our design, gSAMURAI.

We next verified the cellular characteristics and potency of gSAMURAI PGCs. Immunofluorescence revealed that PGC markers such as DAZL, CVH and NANOG were positively stained in gSAMURAI PGCs (Fig. 3E). These results suggested that the characteristics of PGCs were maintained after genetic manipulation and propagation.

### Generation of gSAMURAI chimeric chickens

**Fig. 4.**
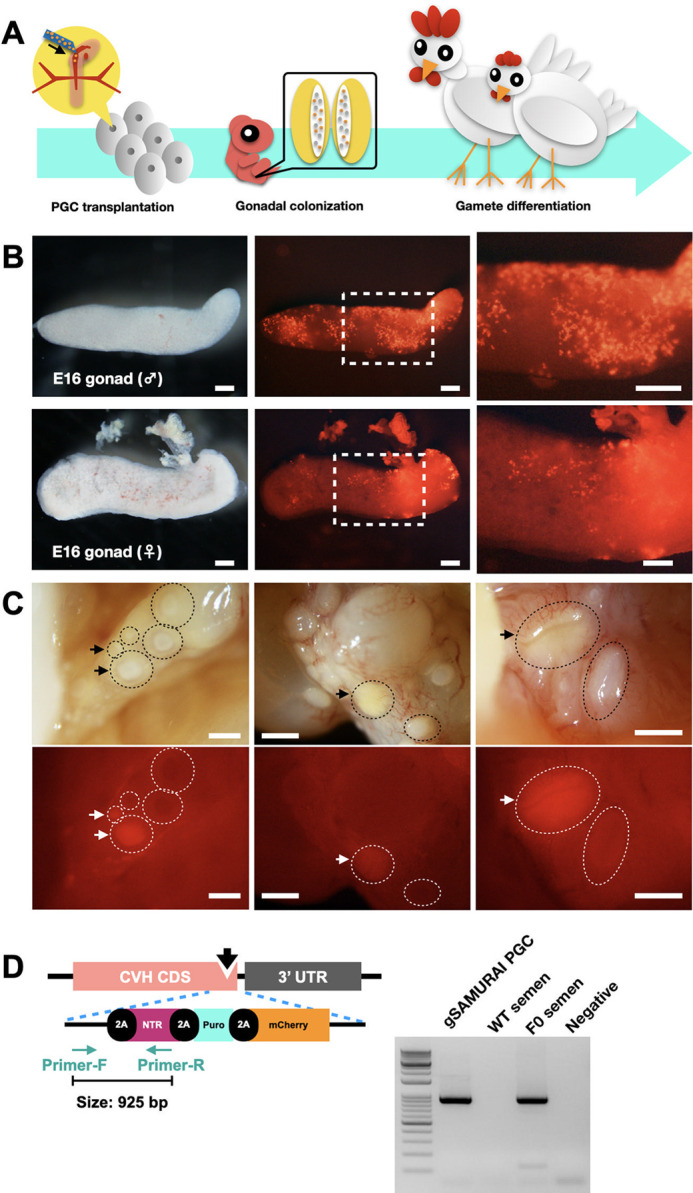
**Characterization and function assay in CVH homologous KI gSAMURAI PGCs.** (A) Flowchart of transplantation as the functional assay for gSAMURAI PGCs. (B) Chick gonads at E16 of incubation were isolated from the PGC-transplanted embryos to visualize mCherry fluorescence on transplanted cells. Dashed boxes indicate the area enlarged on the right. (C) Follicles of the ovary dissected from PGC-transplanted F0 hens showed mCherry fluorescence (arrows). Dashed lines encircle follicles. (D) PCR amplification of the DNA fragment (925 bp) crossing the endogenous CVH CDS to the transgene region in the DNA samples from gSAMURAI PGCs, WT rooster semen, and PGC-transplanted F0 rooster semen. The DNA-free reaction is shown as a negative control. Scale bars: 500 µm. Images are representative of more than three samples.

The gSAMURAI germline chimeric chicken was generated by transferring PGCs carrying the gSAMURAI transgene into the blood vessels of E3 embryos (Fig. 4A). Some of the treated embryos were incubated until E16 and sacrificed to confirm the gonadal colonization of gSAMURAI PGCs. In the gonads from both male and female recipients, the mCherry-expressing cell population could be obviously found (Fig. 4B), suggesting that gSAMURAI PGCs retained the ability to migrate via the circulation and colonize the gonads. In the ovary of a 150-day-old hen that was transplanted with gSAMURAI PGCs, several follicles were found to present mCherry fluorescence (Fig. 4C). Semen samples collected from the PGC-transplanted chimeric (F0) roosters were examined for PCR amplification of transgene fragments. The PCR fragment specific for the *CVH* KI allele was detected in DNA samples from gSAMURAI PGCs and F0 rooster semen, but not in those from WT rooster semen (Fig. 4D), confirming that the KI transgene cassette was placed in the *CVH* locus, as designed, in the population of gSAMURAI PGCs and F0 rooster sperm.

### Germline-specific mCherry expression in gSAMURAI offspring allows visualization of germ cells

**Fig. 5.**
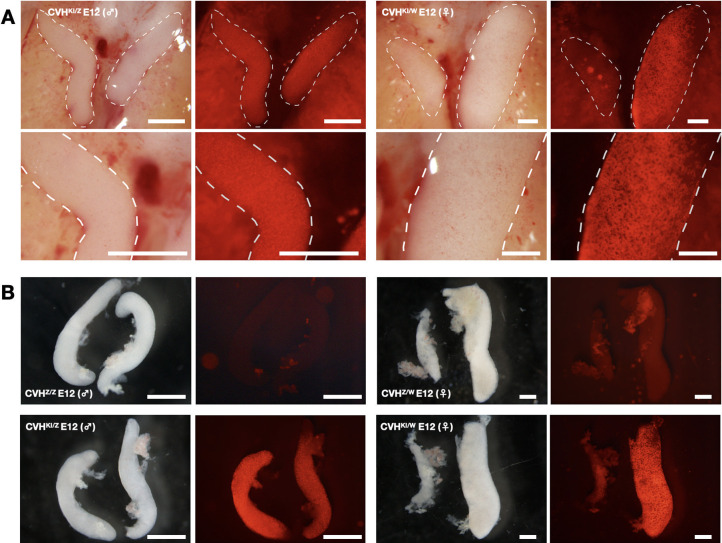
**Germline-specific mCherry expression in gSAMURAI chick embryonic gonads.** (A) Heterozygous gSAMURAI E12 males (CVH^KI/Z^) and females (CVH^KI/W^) showed mCherry expression in germ cells of gonads (white dashed line). (B) Dissociated gonads from WT chick embryos presented no fluorescence, in contrast to the brilliant mCherry expression in gonads isolated from gSAMURAI chick embryos. Scale bars: 250 µm. Images are representative of more than three samples.

We generated gSAMURAI offspring by intercrossing the germline chimeric rooster (F0) to WT hens with artificial insemination. To confirm inheritance of the gSAMURAI transgene, F1 embryos were dissected at E12 (Fig. 5A). Seven out of 37 (18.9%) embryos carried the gSAMURAI transgene, and these embryos presented mCherry expression in germ cells of both site gonads in both sexes, whereas comparatively few mCherry-expressing cells were found in the right site gonad compared with the left in the female. This is consistent with the nature of asymmetry between the left and right gonads in gonadogenesis; the left side shows a larger number of germ cells, especially in the female gonad (Intarapat and Stern, 2013). We isolated both gonads from WT and gSAMURAI embryos (Fig. 5B). No fluorescent cells were found in the WT gonads regardless of sex. By contrast, gSAMURAI embryonic gonads showed robust mCherry expression in both gonads of the male (CVH^KI/Z^) and the dominant gonad (right) of the female (CVH^KI/W^).

We also observed the fluorescent germ cells of gSAMURAI embryos at E2 with an *ex ovo* embryo culture. gSAMURAI germ cells expressing mCherry were observed circulating in the blood vessels at E2.5 [Hamburger–Hamilton stage (HH) 13-14], and a small proportion of them arrested at the area between two bilateral dorsal aortae to microvessels and near the region for the genital ridge (Movie 1). This phenomenon continued at the E3 stage. Germ cell efflux from the dorsal aorta was also observed, which stopped in small capillaries (Movies 2, 3). The area with arrested germ cells was enlarged to cover almost all the outer sides of the bilateral dorsal aortae behind the tail bud and condensed in the region of the genital ridge (Movie 4) (Saito et al., 2022).

We succeeded in generation of F1 gSAMURAI chickens from F0 chimeras, and also produced F2 gSAMURAI offspring from the F1 at the Mendelian rate, which showed fluorescence in germ cells. These results demonstrated that the genome-edited gSAMURAI PGCs are inherited by subsequent generations and that the gSAMURAI transgene does not affect development, fertility or inheritance. Additionally, the resulting gSAMURAI offspring exhibited germ cell-specific fluorescence, enabling the tracking of germ cells in live embryos.

### Ablation of gSAMURAI PGCs by administering Mtz enriches exogenously transplanted PGCs

**Fig. 6.**
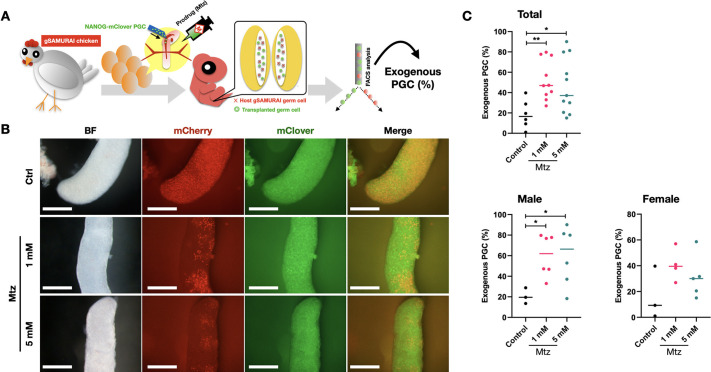
**Prodrug-Mtz supplementation with PGC transplantation in gSAMURAI recipients.** (A) Schematic of the Mtz administration test using gSAMURAI chick embryos as a recipient to compare the proportion of exogenous PGCs after PGC transplantation. (B) E10 gonads dissociated from NANOG-mClover PGC-transplanted gSAMURAI embryos with different treatments. Scale bars: 250 µm. Images are representative of more than three samples. (C) Quantification of the exogenous PGC rate derived by FACS analysis in different treatment groups. *P<0.05; **P<0.01 (one-way ANOVA with LSD test; *n*=27). Horizontal line represents mean.

We examined whether inducible germline-specific cell ablation works in gSAMURAI chickens. First, we administered a prodrug of the NTR/Mtz system, Mtz, when transplanting exogenous PGCs into gSAMURAI embryos. To distinguish the exogenous PGCs from gSAMURAI PGCs, PGCs expressing mClover under the control of chicken *NANOG*, a gene expressed in PGCs, were utilized as donor PGCs (Fig. S1). As shown in Fig. 6A, we transplanted NANOG-mClover PGCs into gSAMURAI E3 embryos together with different dosages of Mtz and incubated these embryos until E10. The viability of the E10 embryos after transplantation was 83.3% (10/12 embryos) in the control group, 76.2% (16/21 embryos) and 81.8% (18/22 embryos) in the Mtz-1 mM and Mtz-5 mM groups, respectively, and there was no difference in viability between gSAMURAI and WT embryos (Tables S3 and S4).

Fig. 6B shows the colonization of gSAMURAI and NANOG-mClover PGCs in gonads. In the non-prodrug control group, both transplanted NANOG-mClover PGCs and host gSAMURAI PGCs shown by mCherry expression were found in the gonads in a well-distributed manner. By contrast, in the Mtz administration groups, the occupancy of gSAMURAI PGCs in gonads was obviously reduced, whereas transplanted NANOG-mClover PGCs were well distributed. Quantification (Fig. 6C) revealed that the transplanted PGC rate in the total recipients of the Mtz administration groups was significantly higher than that of the control group (*P*<0.05). Furthermore, the quantified data were scattered by the sex of recipients. Female recipients in the Mtz administration groups showed higher average exogenous PGC rates than those in the control group, but no significant difference was found. In contrast, among male recipients, the exogenous PGC rate in both doses of Mtz administration groups was significantly higher than that of the control group (*P*<0.05). Moreover, all higher exogenous PGC rates (>70%) in the Mtz administration groups were found in males. The highest exogenous PGC rate was 28.77% in the control group, and the highest rates were 79.67% and 90.15% in the Mtz-1 mM and Mtz-5 mM groups, respectively.

These results indicated that the NTR/Mtz system functions to ablate the host germ cells in gSAMURAI embryos without affecting exogenous PGC migration and colonization. An extreme predominance in the donor germ cell population was generated by using the gSAMURAI chicken model as the recipient, indicating a largely increasing potential to obtain the target offspring after sex maturity.

## DISCUSSION

Recently, several studies reported the production of gene-edited chickens using transcription activator-like effector nucleases (TALENs) or the CRISPR/Cas9-mediated method (Oishi et al., 2016; Park et al., 2014). In this study, we successfully generated a genetically modified chicken with the CRISPR-Cas12a (Cpf1) family nuclease MAD7. MAD7 differs from Cas9 in its nuclease recognition sequence and the DNA end sequence after cleavage. MAD7 recognizes a thymidine-rich sequence in the PAM site upstream of the crRNA, whereas Cas9 uses a guanine-rich PAM sequence downstream of the crRNA. crRNAs of Cpf1 family nucleases, including MAD7, are shorter than Cas9 single guide RNAs (sgRNAs) owing to differences in repeat- and tracrRNA-derived segments. The Cpf1 family crRNA is only ∼43 nt in length, whereas the most commonly used sgRNA scaffold for Cas9 is ∼101 nt in length (Swarts and Jinek, 2018). Concerning the DNA end sequence after cleavage, MAD7 uses a single RuvC-like endonuclease domain to cut each DNA strand to form a sticky end, whereas Cas9 uses HNH and RuvC nuclease domains to form a blunt-ended DSB (Swarts and Jinek, 2018). The two different types of gene-editing nucleases allow for a wider range of target designs.

In the present study, we demonstrated that MAD7 introduced a mutation into the *CVH* loci of chicken PGCs at ∼10% indel frequency. Compared with the indel formation from 4% to 23% found in a human cell line when MAD7 was used, it was in the range of prediction (Liu et al., 2020). By using this nuclease for DNA cleavage-mediated homologous recombination, gene KI in the *CVH* loci of chicken PGCs was achieved. Following antibiotic selection for 2 weeks, almost 100% of KI PGCs could be obtained without affecting PGC characteristics. MAD7 shows sufficient enzymatic activity in the genetic modification among chicken PGCs as other nucleases.

We generated gSAMURAI chickens expressing a germline-specific mCherry reporter, which will be a powerful tool in the study of germ cell development. *CVH* was used as the leading gene for the reporter. A recent study also utilized *CVH* as the KI target gene to visualize PGCs (Ezaki et al., 2022). CVH/DDX4 is a metazoan-conserved protein that encodes an RNA-binding protein and plays an important role in germline formation (Mochizuki et al., 2001). Its protein expression has been detected in the embryo as early as the first cleavage stage, localizing in the cytoplasm of germ cells covering PGCs in the embryo to gametes in adult gonads (Tsunekawa et al., 2000). Avian species are known to translocate PGCs from the extra-embryonic region to gonads through the circulation after being enveloped in vascular tissue, which is different from the mammalian case (Murai et al., 2021; Nakamura et al., 2013). In the present study, using *ex ovo* culture of E2.5-E3 gSAMURAI embryos, we detected mCherry-expressing PGCs circulating in and exiting from blood vessels. PGCs were found to migrate within a regular pattern such that the paired dorsal aorta, accelerated by a heart pump, carried PGCs to enter the tributary stream – the capillary plexus around the genital ridge (Movies 1-4). During this period, the velocity of the PGCs seemed to reduce, and some PGCs became stuck in the capillary and arrested the flow. It has recently been shown that PGC occlusion in the extravasation vascular plexus is a crucial step for further transmigration into the gonad (Saito et al., 2022), suggesting that actin-mediated regulation of cellular stiffness is highly dynamic in avian PGCs during migration. This extravasation event in avian PGCs could be linked to cancer metastasis as they shared a similar cellular migration manner (Reymond et al., 2013). Our gSAMURAI chicken embryo would be useful for such investigations because PGCs are easy to track in this model.

In this study, we demonstrated the efficient ablation of host germ cells using the NTR/Mtz system in a gSAMURAI chicken model. NTR is able to undergo electron transfer from NADH or NADPH to induce a nitro reduction reaction, and cytotoxic alkylating agents are generated when reacting with Mtz, which can cause cell death via the caspase-3-mediated apoptosis pathway (Chen et al., 2011). The composition of gonadal occupancy between the endogenous and exogenous germ cells was changed dramatically by treatment with the prodrug: endogenous germ cells became the minority population, and exogenous ones displayed the dominant roles and constituted the highest occupancy (approximately 90%) among samples. This result indicates that the gSAMURAI model could be an ideal recipient of germ cell transplantation for germ plasm management and/or new strain development in terms of saving time and cost. Regarding the sensitivity of the NTR/Mtz system, prodrug administration at a higher concentration (∼10 mM) and with enough exposure time (24 h) were required to deplete NTR-expressing specific cell types in zebrafish (Curado et al., 2007; Hu et al., 2010). However, the administration method was completely different in the fish and chicken models. In our study, the prodrug was directly injected into embryonic circulation instead of being exposed to the living environment, as in fish models. Recently, the Mtz chemical analogs furazolidone and ronidazole were found to be effective at lower working concentrations with NTR, especially ronidazole, which showed more potential as a replacement for Mtz owing to its lower nonspecific toxicity (Lai et al., 2021). Therefore, for a more consistent result with high efficiency of target cell depletion, the optimum type, method, and exposure period of prodrug administration still needs further investigation.

The gSAMURAI chicken model has advantages compared with classical methods. In classical strategies, host-derived germ cells have been partially removed by chemical or irradiation treatment, but these strategies resulted in high embryonic mortality and/or abnormality (Nakamura et al., 2012, 2010). Although our gSAMURAI chicken requires administration of the prodrug Mtz to remove germ cells, ∼80% of embryos administered the prodrug developed normally until E10, which is comparable to the control group without prodrug. This indicates that Mtz administration does not affect the survival rate and embryo development. In addition, the ablation of gSAMURAI PGCs was detected both in males and females. However, there was a slight difference in ablation efficiency. A significant difference was found only in males, and not in females, indicating that differences in prodrug sensitivity might exist between the sexes.

Recently, genetic methods for this purpose were developed to overcome this problem. *CVH* null chickens were generated to show germ cell ablation and thus provided an ideal surrogate host for the generation of avian species by PGC transfer (Taylor et al., 2017). Within this genetically sterile model, an almost perfect transmission rate was obtained in a female recipient with the same sexual PGC lines as donors, whereas the WT recipient female with the identical set of PGC transfers showed no heritage from donor PGCs in the study (Woodcock et al., 2019). Nevertheless, maintenance and production of *CVH* null chickens will require much effort because sterile chickens never produce offspring. Therefore, an inducible sterile model is likely more suitable as the side-effect problem in the previous method could be overcome. Ballantyne et al. (2021) described a genetically modified chicken model produced by a gene insertion resulting in expression of an inducible caspase-9 (iCaspase9) protein driven by the endogenous *DAZL* gene; thus, a switchable germ cell-deficient model was formed. Using this system as a surrogate, they produced chicken offspring carrying edited alleles inherited from donor PGCs with a high germline transmission rate because almost all host germ cells were ablated. However, the iCaspase9 chicken model presents a comparatively low hatchability rate (∼60%), indicating an influence of the iCaspase9 transgene on chicken embryonic development (Ballantyne et al., 2021).

In conclusion, we have generated a genetically modified chicken model, termed gSAMURAI, that provides, not only a convenient method of germ cell tracking as a tool for research on germ cell development, but also an ideal recipient for any production purpose that uses germ cell transfer because endogenous germ cells could be depleted with a simple induction process. This function will facilitate production efficiency and thus will be expected to contribute to avian germ plasm management for the regeneration of endangered species by inter-species germ cell transfer and the production of novel poultry strains with gene-editing techniques.

## MATERIALS AND METHODS

### Chicken and chicken embryos

For PGC donor embryos, Barred Plymouth Rock (BPR) strain chicken (*Gallus gallus*) fertile eggs were purchased from Okazaki station, National Livestock Breeding Center, Japan. For recipient embryos of gSAMURAI PGC transfer, fertile eggs of JuliaLite strain chickens (*Gallus gallus*) were purchased from Japan Layer K.K., Japan. gSAMURAI fertile chicken eggs were obtained by artificial insemination at Kyodoken Institute, Japan. All animal procedures were approved by the Institutional Animal Care and Use Committee of Tokushima University (T2021-53) and Kyodoken Institute (A2021-001-3).

### Plasmid construction

The MAD7 expression plasmid was synthesized using the publicly available sequence of the pX330-U6-Chimeric_BB-CBh-hSpCas9 plasmid (Addgene plasmid #42230; Cong et al., 2013) as a backbone and replacing the regions for a crRNA and a fragment from 3xFLAG to nucleoplasm NLS with a MAD7 crRNA and a cassette containing a C-terminal, NLS-conjugated MAD7 nuclease (nMAD7), respectively. The P2A-mScarlet fragment was obtained by PCR cloning from pEB2-mScarlet (a kind gift from Dr Philippe Cluzel; Addgene plasmid #104006; Balleza et al., 2018) and inserted following the 3xHA by assembling with NEBuilder HiFi DNA Assembly (New England Biolabs). The donor plasmid for homologous recombination was generated by inserting a synthetic cassette containing T2A-NTR-T2A-Puromycin N-acetyltransferase-T2A-mCherry at the location before the stop codon of the *CVH* coding DNA sequence (CDS) with NEBuilder HiFi DNA Assembly. The homology-arm sequence was cloned in a genomic DNA (gDNA) template (chromosome Z: 17501775-17503792, bGalGal1.mat.broiler. GRCg7b) and inserted into the pBluescript II SK (+) plasmid by TA cloning. All synthetic DNA fragments were purchased from Integrated DNA Technologies Inc.

For crRNA cloning, a pair of BbsI enzymatic digestion sites were designed located just after the MAD7 crRNA in the MAD7 expression plasmid for the cloning of annealed crRNA oligos by restriction cloning with BbsI and T4 ligase (New England Biolabs). crRNA candidates were designed with CHOPCHOP (Labun et al., 2019), and oligos were synthesized and purchased from Eurofins Japan.

### Cell culture and manipulation

Donor PGC clones were derived from E3 BPR strain chick embryos and maintained with the method described by Chen et al. (2019). The derived PGC clones were stored at −80°C within Bambanker freezing medium (NIPPON Genetics) for further usage. The culture medium for PGC was composed of a basic medium and supplements (Table S1). For genetic modification in PGCs, 1 µg of plasmid DNA was used for 5×10^4^ cells in a 10 µl electroporation reaction. For KI purposes, the ratio of MAD7 expression plasmid and donor plasmid was 1:2 (w/w). Electroporation was conducted with an optimized parameter of a 1150 V/30 ms/1 pulse with buffer R in a Neon Transfection System (Thermo Fisher Scientific). The PGC culture medium contained puromycin (0.1 µg/ml; Sigma-Aldrich), but no other antibiotics were used for puromycin selection (Chen et al., 2018).

### FACS and analysis

For fluorescent cell enrichment sorting and analysis, experiments were conducted using a BD FACSMelody cell sorter (BD Biosciences) with the default configuration and equipped with blue (488 nm), red (640 nm), yellow-green (561 nm) lasers and BD Chorus software (BD Biosciences). Instrument operation was performed according to the manufacturer's instructions.

### DNA extraction and amplicon sequence analysis

gDNA samples from cells were prepared with a DNeasy Blood & Tissue Kit (QIAGEN). The DNA fragments around the crRNA target site were amplified by two-step PCR with specific primer sets (Table S2) and Index PCR Primers described in the manufacturer's instructions (Illumina). After gel purification, the amplicons were subjected to MiSeq for amplicon sequence analysis using the MiSeq Reagent Kit v2 (Illumina).

### Immunofluorescence staining

Cells were attached to a hydrophilic micro slide glass (Matsunami Glass) and adapted to immunofluorescence staining following methods described previously (Chen et al., 2019). Cells were labeled with anti-DAZL (Abcam, AB215718; 1:200), anti-CVH (anti-DDX4; Bioss, BS-3597R; 1:200), anti-NANOG (a kind gift from Dr Kiyokazu Agata; 1:1000; Nakanoh et al., 2015) and anti-mCherry (Novus Biologicals, NBP1-96752; 1:500) antibodies and then subsequently stained with Alexa Fluor™ 488-conjugated donkey anti-rabbit IgG (Invitrogen, A-21206; 1:2000) and Alexa Fluor™ 555-conjugated donkey anti-mouse IgG (Invitrogen, A-31570; 1:2000) for visualization. Hoechst 33342 (2.5 µg/ml; Molecular Probes) was used to stain the nucleus. Images were acquired using a Leica DMI4000 B microscope (Leica Microsystems) equipped with a digital CMOS camera (Hamamatsu Corporation).

### Chicken embryo culture and dissection

*In ovo* chicken embryos were incubated in a humidified egg incubator (Showafuranki) at 38°C, and automatic egg turning was performed. For *ex ovo* embryo culture, E2.5 embryos were excised from the yolk and cultured on a Petri dish following a method described by Uchikawa et al. (2004) for observation. For embryo dissection, the embryo was isolated at E10, E12 or E16. Gonads were collected by gently picking them up with tweezers and then washing them once in cold PBS without Ca^2+^/Mg^2+^ (Takara Bio). Image and movie acquisition was conducted using a Leica M205 FA fluorescence histological microscope (Leica Microsystems) equipped with cellSens Standard (Olympus).

To prepare the sample for FACS analysis, gonad tissue was dispersed in TrypLE™ Express Enzyme (Gibco) with gentle shaking for 10 min. After centrifugation (600 ***g***, 5 min), dispersed tissue was resuspended in PBS without Ca^2+^/Mg^2+^ and passed through a 100 µm strainer (Falcon) for further analysis.

### PGC transplantation and prodrug supplementation

A total of 5×10^4^ PGCs were transplanted into the circulation of E3 recipients in 5 µl of a basic medium or prodrug-containing medium following the method described by Chen et al. (2019) for the gonad migration assay. Prodrug-containing groups were prepared just before the transplantation by diluting some volume of cell suspension and the basic medium of Mtz at 2 mM or 10 mM to obtain a final concentration of 1 mM or 5 mM, respectively. Mtz was purchased from Fujifilm Wako Pure Chemical Corporation. Data were analyzed with GraphPad Prism9 (GraphPad Software, Inc.) using one-way analysis of variance (ANOVA) and Fisher's least significant difference (LSD) test. Differences between groups were considered to be significant at a *P*<0.05 or P<0.01.

## Supplementary Material

10.1242/develop.202079_sup1 Supplementary information
