# Supplementary material for: An inducible germ cell ablation chicken model for high-grade germline chimeras
Source: Development. 2023 Sep 25;150(18):dev202079. doi: 10.1242/dev.202079 (PMC10560566; doi:10.1242/dev.202079)
Supplement: Supplementary information [file develop-150-202079-s1.pdf]

Fig. S1. Chen et al.

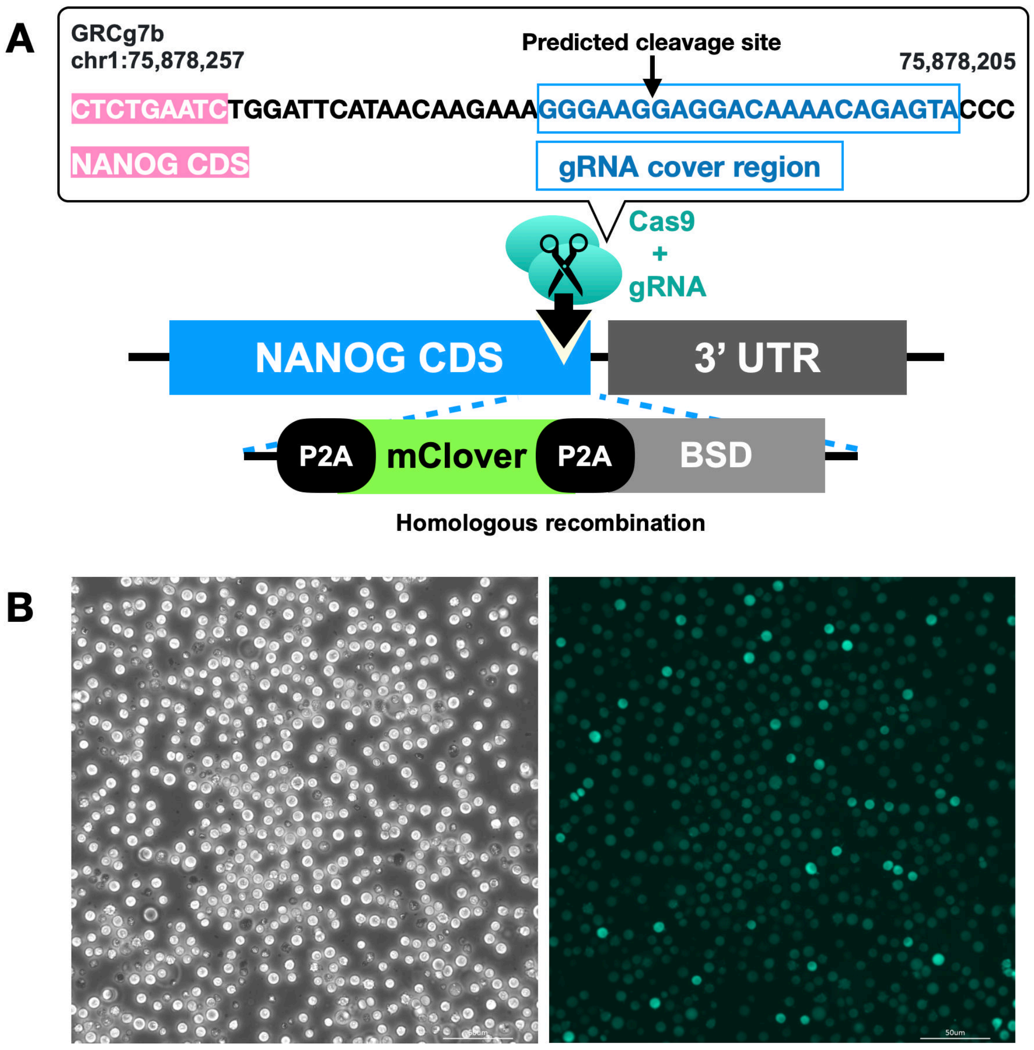

Fig. S1. Generation of NANOG-mClover PGC line.

(A) NANOG-mClover PGC line was generated by inserting the mClover fluorescent protein CDS into the 3' end of the chicken Nanog coding sequence via the 2A peptide sequence using the CRISPR/Cas9 system. The target sequence of guide RNA is 5'-ATGAGACAAAACAGGAGGAAGGG-3, which corresponds to 75878208-75878230, NC_052532.1, bGalGal1.mat.broiler.GRCg7b. The donor plasmid carries mClover and Blasticidin S Deaminase (BSD) sequences flanked by 1kb long homology-arms. (B) Fluorescence of NANOG-mClover PGCs (Scale bar: 50 µm).

Development • Supplementary information

Table S1. Culture media for PGC culture

Component	Manufacture	Identifier	Concentration
Basic medium			
Ca^{2+} free DMEM	nacalai tesque, Japan	16972-45	As the base*
Sterile water	FUJIFILM Wako Pure Chemical Corporation, Japan	039-24155	As the base*
Nucleosides	Merck, Germany	ES-008-D	1X
GlutaMAX	Gibco, USA	35050061	2mM
Pyruvate	Gibco, USA	11360070	1mM
NEAA	Gibco, USA	11140050	1X
Beta-mercaptoethanol	Gibco, USA	21985023	0.1mM
Antibiotics**	FUJIFILM Wako Pure Chemical Corporation, Japan	161-23181	1X
Supplements			
B-27 supplement w/o VitA	Gibco, USA	12587001	1X
Ovalbumin	Sigma-Aldrich, USA	A5503	0.2% (2mg/ml)
Heparin	nacalai tesque, Japan	17513-54	0.1mg/ml
Ovotransferrin	Sigma-Aldrich, USA	C0755	10μg/ml
$CaCl_2$	nacalai tesque, Japan	06731-05	0.15mM
Activin A	R&D Systems, USA	338-AC	25ng/ml
FGFC	FUJIFILM Wako Pure Chemical Corporation, Japan	067-06591	4ng/ml

* The base was prepared with the ratio 3:1 (v/v) between DMEM and water, respectively.

** No presence of antibiotics in the medium for electroporation

Development • Supplementary information

Table S2. PCR primer sets

Primer name	Sequence (5’->3’)	Product length (bp)	Note
YCC-VASA_NGS_F1	TCGTCGGCAGCGTCAGATGTGTATAAGAGACAGGCAGTTATAGAAAGCTCTTTCCTTGT	351	cVASA amplicon sequence analysis (Fig. 1)
YCC-VASA_NGS_R1	GTCTCGTGGGCTCGGAGATGTGTATAAGAGACAGCAACAGTTCACTTAAACCACAACAG	351	cVASA amplicon sequence analysis (Fig. 1)
cVasa PCR F1	AGCATGTTCATTTTAGACTGGAGTC	2559; 423	Transgene insertion (Fig. 2)
cVasa PCR R1	ATCAACGATGGGTTCCTTTTTCATT	2559; 423	Transgene insertion (Fig. 2)
cVasa PCR F1	AGCATGTTCATTTTAGACTGGAGTC	925	Transgene genotyping (Fig. 3)
NTR-genotyping-R-2	AGGGTAGCGTTGAAGTCCTC	925	Transgene genotyping (Fig. 3)

Table S3. The viability at E10 after PGC transplantation with or without Mtz administration

Group	Recipient embryos*	Survival embryo at E10	Viability (%)	The genotype of survival embryos	
Control	12	10	83.3	gSAMURAI	6
				WT	4
Mtz-1mM	21	16	76.2	gSAMURAI	10
				WT	6
Mtz-5mM	22	18	81.8	gSAMURAI	11
				WT	7

*All embryo for the transplantation was produced by crossing gSAMURAI males ($CVH^{KI/Z}$) with gSAMURAI females ($CVH^{KI/W}$) or WT females ($CVH^{Z/W}$).

Table S4. Quantification of the exogenous PGC rate derived by FACS analysis in different treatment groups

Group	Sample#	Host sex	Fluorescent cell number		Exogenous PGC (%)	Genotype
			mCherry	mClover		
Control	1	M	2134	862	28.77	$CVH^{KI/Z}$
	2	F	6127	62	1.00	$CVH^{KI/W}$
	3	M	2498	607	19.55	$CVH^{KI/Z}$
	4	F	5424	556	9.30	$CVH^{KI/W}$
	5	M	2671	417	13.50	$CVH^{KI/Z}$
	6	F	4793	3151	39.67	$CVH^{KI/W}$
Mtz-1 mM	1	F	1262	465	26.93	$CVH^{KI/W}$
	2	M	99	388	79.67	$CVH^{KI/KI}$
	3	F	802	557	40.99	$CVH^{KI/W}$
	4	M	819	720	46.78	$CVH^{KI/Z}$
	5	M	28	25	47.17	$CVH^{KI/KI}$
	6	M	603	2100	77.69	$CVH^{KI/KI}$
	7	M	42	139	76.80	$CVH^{KI/Z}$
	8	F	895	551	38.11	$CVH^{KI/W}$
	9	M	1044	513	32.95	$CVH^{KI/Z}$
	10	F	733	972	57.01	$CVH^{KI/W}$
Mtz-5 mM	1	M	1178	1322	52.88	$CVH^{KI/KI}$
	2	M	321	1404	81.39	$CVH^{KI/KI}$
	3	M	408	1626	79.94	$CVH^{KI/Z}$
	4	M	1516	895	37.12	$CVH^{KI/Z}$
	5	F	1679	433	20.50	$CVH^{KI/W}$
	6	F	2000	353	15.00	$CVH^{KI/W}$
	7	F	763	328	30.06	$CVH^{KI/W}$
	8	M	1522	341	18.30	$CVH^{KI/Z}$
	9	F	502	712	58.65	$CVH^{KI/W}$
	10	F	747	346	31.66	$CVH^{KI/W}$
	11	M	125	1144	90.15	$CVH^{KI/Z}$

Development • Supplementary information

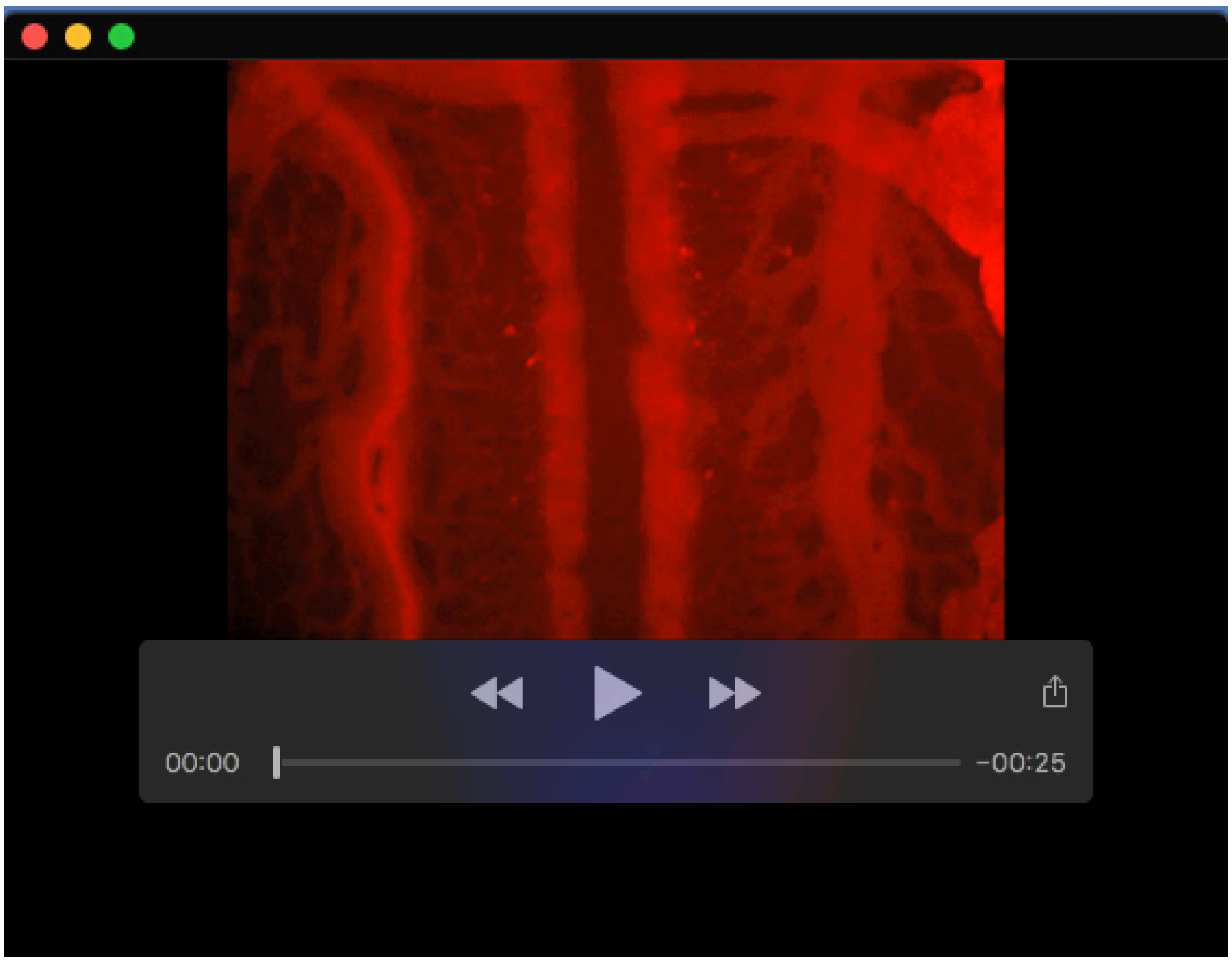

Movie 1. The germ cell circulation at the region surrounding the genital ridge of an ex-ovo cultured E2.5 gSAMURAI embryo.

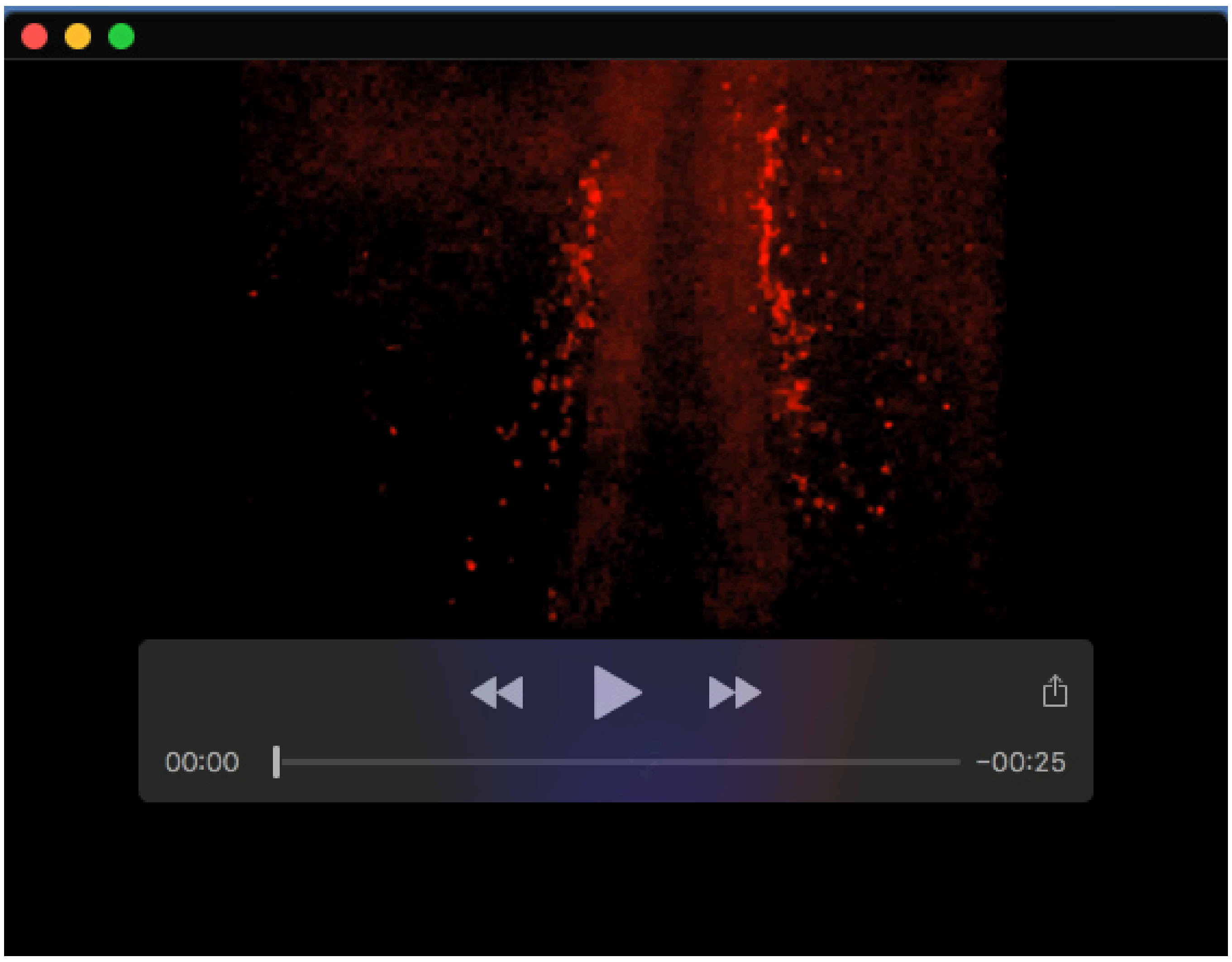

Movie 2. The germ cell circulation at the region surrounding the genital ridge of an ex-ovo cultured E3 gSAMURAI embryo.

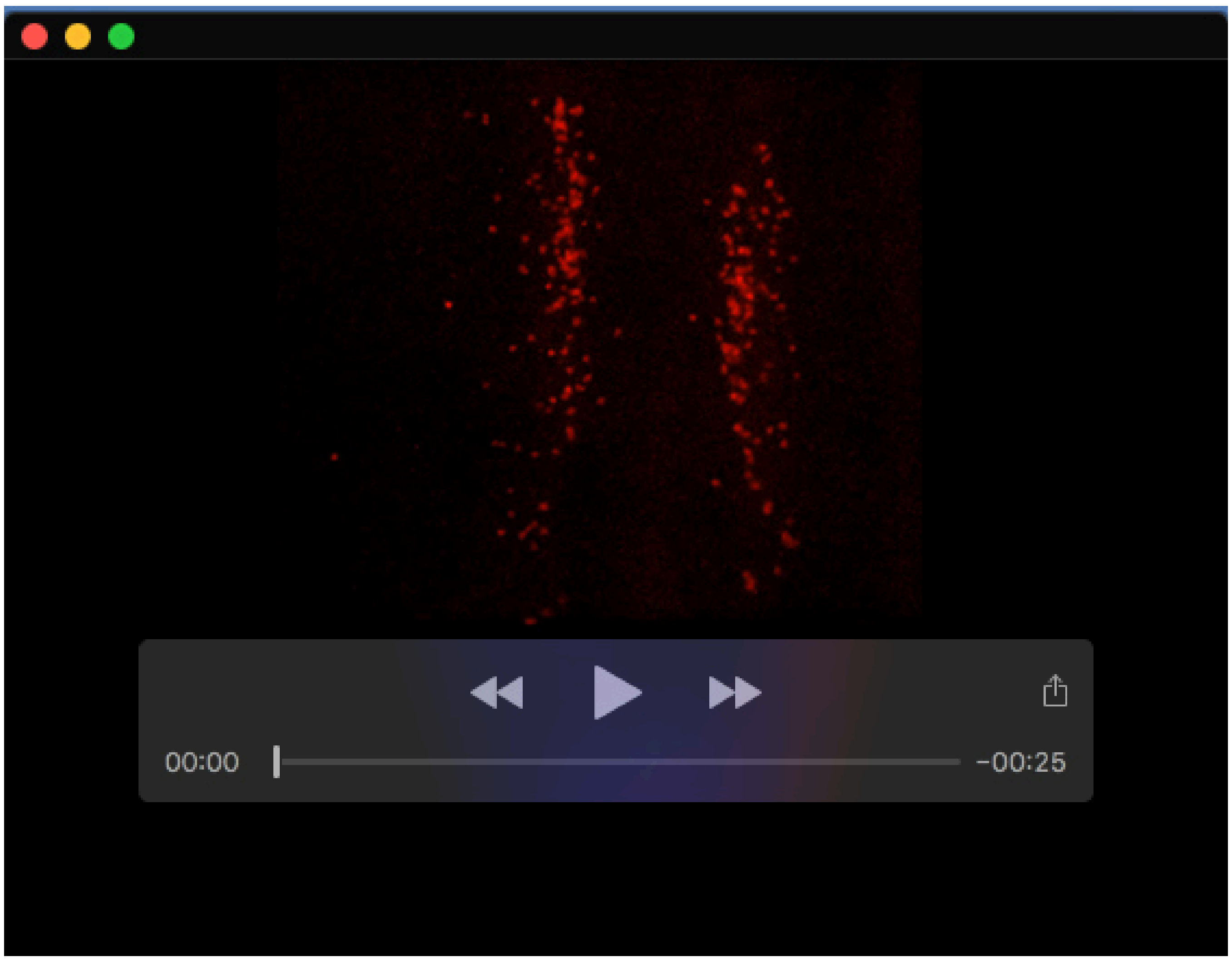

Movie 3. Migrating germ cells arrested at small capillaries when effluxed from the dorsal aorta to capillaries in E3 gSAMURAI embryos.

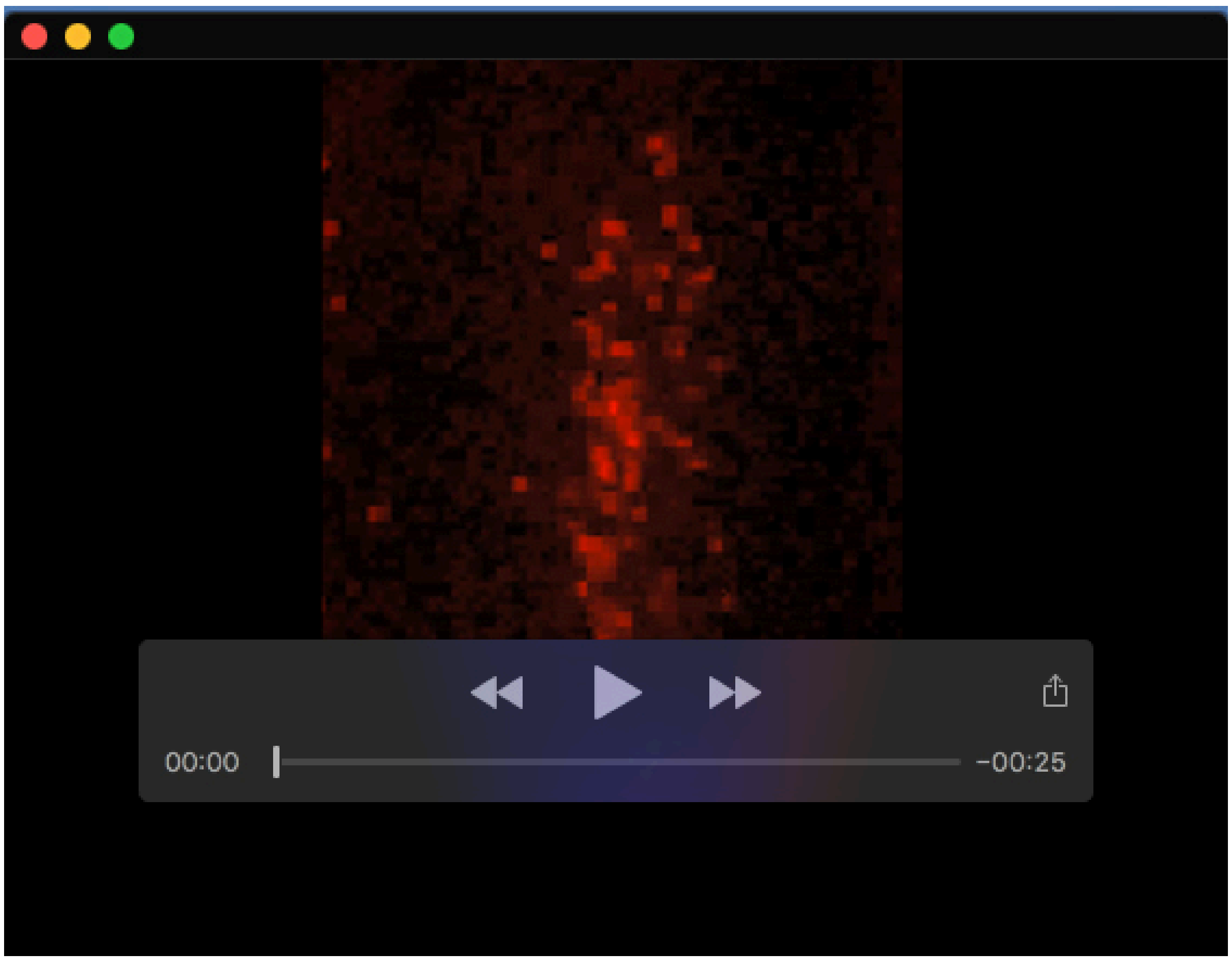

Movie 4. An enlarged view and arrow labels migrating germ cells arrested at small capillaries in E3 gSAMURAI embryos.

Development • Supplementary information
