# Supplementary material for: An inducible germ cell ablation chicken model for high-grade germline chimeras
Source: Development. 2023 Sep 25;150(18):dev202079. doi: 10.1242/dev.202079 (PMC10560566; doi:10.1242/dev.202079)
Supplement: Supplementary file 1 [file develop-150-202079_review_history.pdf]

An inducible germ cell ablation chicken model for high-grade germline chimeras

Yi-Chen Chen, Daisuke Saito, Takayuki Suzuki and Tatsuya Takemoto
DOI: 10.1242/dev.202079

Editor: Liz Robertson

Review timeline

Original submission:	14 June 2023
Editorial decision:	14 July 2023
First revision received:	3 August 2023
Editorial decision:	22 August 2023
Second revision received:	22 August 2023
Accepted:	24 August 2023

Original submission

First decision letter

MS ID#: DEVELOP/2023/202079

MS TITLE: An inducible germ cell ablation chicken model for high-grade germline chimeras

AUTHORS: Yi-Chen Chen, Daisuke Saito, Takayuki Suzuki, and Tatsuya Takemoto

I have now received all the referees' reports on the above manuscript, and have reached a decision. The referees' comments are appended below, or you can access them online: please go to BenchPress and click on the 'Manuscripts with Decisions' queue in the Author Area.

As you will see, the referees express considerable interest in your work, but have some significant criticisms and recommend a substantial revision of your manuscript before we can consider publication. If you are able to revise the manuscript along the lines suggested, which may involve further experiments, I will be happy receive a revised version of the manuscript. Your revised paper will be re-reviewed by one or more of the original referees, and acceptance of your manuscript will depend on your addressing satisfactorily the reviewers' major concerns. Please also note that Development will normally permit only one round of major revision. If it would be helpful, you are welcome to contact us to discuss your revision in greater detail. Please send us a point-by-point response indicating your plans for addressing the referee's comments, and we will look over this and provide further guidance.

Please attend to all of the reviewers' comments and ensure that you clearly highlight all changes made in the revised manuscript. Please avoid using 'Tracked changes' in Word files as these are lost in PDF conversion. I should be grateful if you would also provide a point-by-point response detailing how you have dealt with the points raised by the reviewers in the 'Response to Reviewers' box. If you do not agree with any of their criticisms or suggestions please explain clearly why this is so.

© 2023. Published by The Company of Biologists under the terms of the Creative Commons Attribution License (https://creativecommons.org/licenses/by/4.0/).

Reviewer 1

Advance summary and potential significance to field

The development of a new 'universal' recipient model for PGCs cells edited in the chicken model is an asset for the entire avian field. This can help to validate the role of genes in many research fields and facilitate the dissemination of these GE approaches in the avian field.

Comments for the author

In the paper entitled «An inducible germ cell ablation chicken model for high-grade germline chimeras » the authors, Chen et al., describe a novel model to get a 'less' fertile surrogate chicken that could be used for producing GE chicken by introducing chicken PGCs in this new model. As a general comment, the manuscript is 'almost perfect' and outstanding clear and only few changes have to be made, mainly to be more precise on very few points
- In introduction, the authors should mention and add several references of the existing models of sterile surrogate chicken that were already developed by few laboratories (UK, Czech R, Hungary, etc..). Some of these references are indeed mentioned in the discussion – too long- , but should be put in the introduction. This would help to better see the interest of developing other complementary models

Minor comments:
- Line 350, NANOG is not a « specific » PGC marker gene, please change the wording
- Line 425, just mention the reference and supress 'by our research partners' as associated to the manuscript.

Additional comments:
The elements are gathered to validate this new beautiful model, but the efficiency and the frequency of obtaining this progeny is not exemplified. No data are given on the viability of the GE modified line obtained, both in terms of performances of reproduction and use of the 'potential' eggs recipients of edited PGCs (fertility, genotype, robustness of the injected embryos, etc.). This would be an important element for the use and development of this new methodology which seems less difficult to use than the few lines previously obtained to obtain a "sterile" environment to favor exogenous cells. It could be indeed more highlighted in the discussion part. Could the authors bring some elements on the edited chicken line?

The key question, which is NOT demonstrated in the manuscript, remains the efficiency of gonad colonization until the hatching of the chimeras generated by the injection of exogenous PGCs carrying a particular modification or genotype into the modified induced recipients. What are the rate of modified chicken able to pass the modification to the next generation. This is the key point!
The line is just established ... the authors should just be more careful about the stability of the chicken line. Additionnally, is there a difference between male and female in this chemical 'sterilization'.. it is not mentioned at all as to a difference between male and female. Can the authors add data on this point or at least comment? Do they favor the only male line?

Even with these doubts, the manuscript is particularly important in the field of targeted genetic modifications in avian species, especially chicken ... What about quail, whose generation time is much shorter than that of chickens. Could this be extrapolated to other avian species? At least mention in the discussion?
In conclusion, the manuscript is remarkable and deserves to be published subject to the few points raised.

Reviewer 2

Advance summary and potential significance to field

The authors present an inducible system to ablate germ cells in chickens (gSAMURAI). The authors developed this approach to circumvent germ cell competition upon entry of genome edited germ

© 2023. Published by The Company of Biologists under the terms of the Creative Commons Attribution License (https://creativecommons.org/licenses/by/4.0/).

cells into the developing chicken embryo. By selectively ablating the host germ cells, edited germ cells are able to colonise the gonad and propagate unencumbered in future crosses. The generation of these sterile host birds involves the introduction of the bacterial nitroreductase (NTR) gene to the germ-cell specific chicken vasa homolog (CVH) gene locus. Upon administration of metronidazole (Mtz), cytotoxic alkylating agents are produced by NTR, resulting in germ cell apoptosis. Additionally, their gSAMURAI system also carries an mCherry reporter separated by a 2A ribosome skipping sequence, in addition to the NTR (similarly separated), between the CVH coding sequence and its 3'UTR. This results in mCherry fluorescence within the germ cells, in addition to susceptibility to ablation by Mtz introduction.

The authors confirmed the identity of the germ cells edited to carry this gSAMURAI system was unperturbed by staining for usual germ cell markers. They then produced male and female chicks carrying gSAMURAI germ cells within their gonads, and these became adult hens and roosters with propagation in their follicles and semen respectively. These adult birds were crossed to generate both male and female gSAMURAI founders with mCherry fluorescent germ cells. Upon administration of Mtz, and introduction of donor NANOG-mClover PGCs, the authors report an increase of donor PGC occupancy from ~30% in wildtypes to ~80-90% in gSAMURAI birds.

This work presents a new system / line for the selective ablation and visualisation of germ cells in chickens. No biological question or developmental mechanism is investigated here.

Comments for the author

Unfortunately, work previously published in Development in 2017 reported the generation of sterile host chickens (https://doi.org/10.1242/dev.145367) in which a genetic ablation of DDX4 resulted in a total absence of PGCs in the post hatch ovary, and therefore total sterility. This system has been used to generate novel genome edited lines through the introduction of genome edited PGCs (https://doi.org/10.1073/pnas.2020909118), revealing new biological findings, and also to revive rare breeds of chicken (https://doi.org/10.1073/pnas.1906316116). The work presented here is an alternative drug-inducible approach to an available and more efficient technique.
While the inclusion of an mCherry fluorescent reporter into the CVH locus to report on PGC distribution could be of some use potentially to projects interested in early PGC development, it should be noted that work previously published in 2022 reported the generation of a GFP knock-in to the CVH locus to track PGCs in chickens (https://doi.org/10.2141/jpsa.0210067).

First revision

Author response to reviewers' comments

Point-to-Point Response to Reviewers

I appreciate the reviewers for their valuable comments and suggestions.
According to them, I have now revised the manuscript by adding or rephrasing the text. My responses to several questions raised by the reviewers are itemized below.

Reviewer #1:
1. In introduction, the authors should mention and add several references of the existing models of sterile surrogate chicken that were already developed by few laboratories (UK, Czech R, Hungary, etc..). Some of these references are indeed mentioned in the discussion – too long- , but should be put in the introduction. This would help to better see the interest of developing other complementary models
> I added the references (lines 91-106) about the existing surrogate chicken models including classical (irradiation) and genetically modified strategies.

2. Line 350, NANOG is not a « specific » PGC marker gene, please change the wording

© 2023. Published by The Company of Biologists under the terms of the Creative Commons Attribution License (https://creativecommons.org/licenses/by/4.0/).

> I changed the wording about NANOG as the “PGC marker” (line 161, Figure 3 legend) or “a PGC-expressing gene” (line 220).

3. Line 425, just mention the reference and supress ‘by our research partners’ as associated to the manuscript.
> I removed the phrase “by our research partners” (lines 292-293).

Additional comments:
4. The elements are gathered to validate this new beautiful model, but the efficiency and the frequency of obtaining this progeny is not exemplified.
> I have understood the importance of efficiency and frequency of obtaining this progeny, which I speculate to reflect the ratio between the donor and the recipient (gSAMURAI) PGCs in the gonad. We must confirm this by obtaining the progeny. On the other hand, we believe that our gSAMURAI system is very useful at this point for readers who study PGC development. We are willing to distribute gSAMURAI chicken as well as gSAMURAI PGCs to the researchers who want to use it.

5. No data are given on the viability of the GE modified line obtained, both in terms of performances of reproduction and use of the 'potential' eggs recipients of edited PGCs (fertility, genotype, robustness of the injected embryos, etc.).
This would be an important element for the use and development of this new methodology which seems less difficult to use than the few lines previously obtained to obtain a "sterile" environment to favor exogenous cells. It could be indeed more highlighted in the discussion part. Could the authors bring some elements on the edited chicken line?
> I added the sentences in Result (lines 223-226) and Discussion (lines 326-329), and data (Table S3 and S4) describing the viability of gSAMURAI chickens after the prodrug administration, which is comparable to the one of the control group.

6. The key question, which is NOT demonstrated in the manuscript, remains the efficiency of gonad colonization until the hatching of the chimeras generated by the injection of exogenous PGCs carrying a particular modification or genotype into the modified induced recipients. What are the rate of modified chicken able to pass the modification to the next generation. This is the key point!
> I totally agree with the referee’s comment. As I wrote the comment above (4), we will examine the efficiency in the near future.

7. The line is just established ... the authors should just be more careful about the stability of the chicken line.
> We have already produced the F2 gSAMURAI chickens, which are hatched at the Mendelian rate. This indicates that the gSAMURAI transgene does not affect development, fertility, and inheritance. I added this in Results (lines 206-210).

8. Additionally, is there a difference between male and female in this chemical 'sterilization'.. it is not mentioned at all as to a difference between male and female. Can the authors add data on this point or at least comment? Do they favor the only male line?
> I added the sentences (lines 329-333) about the difference between males and females following the prodrug administration. Although the ablation of gSAMURA PGCs was detected both in males and females, there was a slight difference in ablation efficiency.

9. Even with these doubts, the manuscript is particularly important in the field of targeted genetic modifications in avian species, especially chicken ... What about quail, whose generation time is much shorter than that of chickens. Could this be extrapolated to other avian species? At least mention in the discussion?
> Currently, a long-time culture of quail PGCs remains challenging, so genetic modification is not feasible. I suppose the gSAMURAI quail would be established, after the establishment of optimal culture conditions for quail PGCs. But, I would like to avoid describing this possibility as it is beyond the scope of this study.
Another strategy is to transfer the quail PGCs into gSAMURAI chicken. This may be possible according to an earlier study of xenotransplantation between chicken and quail. I added the phrase in line 358.

© 2023. Published by The Company of Biologists under the terms of the Creative Commons Attribution License (https://creativecommons.org/licenses/by/4.0/).

Reviewer #2:
Major Concerns:
1. Unfortunately, work previously published in Development in 2017 reported the generation of sterile host chickens (https://doi.org/10.1242/dev.145367) in which a genetic ablation of DDX4 resulted in a total absence of PGCs in the post hatch ovary, and therefore total sterility. This system has been used to generate novel genome edited lines through the introduction of genome edited PGCs (https://doi.org/10.1073/pnas.2020909118), revealing new biological findings, and also to revive rare breeds of chicken (https://doi.org/10.1073/pnas.1906316116). The work presented here is an alternative drug-inducible approach to an available and more efficient technique.
> I added the recent works about the surrogate chicken models including the papers that reviewer #2 indicated. CVH (DDX4) knockout chicken has advantages and disadvantages as surrogates. CVH knockout chicken (Development in 2017) is sterile and thus perfect as the surrogates, but the frequency to obtain the homozygous knockouts (CVHKO/W) is one-fourth: the mendelian rate to obtain the CVHKO/W by intercrossing CVHKO/Z rooster x CVHZ/W hen. The DAZL-iCaspase9 system is quite similar to our gSAMURAI system, the drug-dependent PGC ablation. Our system will offer an alternative drug-inducible approach.

2. While the inclusion of an mCherry fluorescent reporter into the CVH locus to report on PGC distribution could be of some use potentially to projects interested in early PGC development, it should be noted that work previously published in 2022 reported the generation of a GFP knock-in to the CVH locus to track PGCs in chickens (https://doi.org/10.2141/jpsa.0210067).
> I added the sentence describing this paper (lines 277-279). This study (https://doi.org/10.2141/jpsa.0210067) has demonstrated to visualize the PGCs in chicken, by transferring the PGCs carrying GFP reporter in the CVH locus. This PGC-transferred method is useful for studying PGC development. Indeed, we also utilized this method (Fig. 4, 6, and Fig S1) to visualize the PGCs (gSAMURAI and NANOG-mClover PGCs). However, gSAMURAI chicken line has two advantages compared to this system: (1) All the PGCs are labeled by mCherry in gSAMURAI chicken (only a partial population of the PGCs is labeled by the PGC-transferred method). (2) gSAMURAI chicken can be used to study the PGC induction/emergence from the onset of CVH expression (The PGC-transferred method cannot).

Second decision letter

MS ID#: DEVELOP/2023/202079

MS TITLE: An inducible germ cell ablation chicken model for high-grade germline chimeras

AUTHORS: Yi-Chen Chen, Daisuke Saito, Takayuki Suzuki, and Tatsuya Takemoto

I have now received all the referees reports on the above manuscript, and have reached a decision. The referees' comments are appended below, or you can access them online: please go to BenchPress and click on the 'Manuscripts with Decisions' queue in the Author Area.

The overall evaluation is positive and we would like to publish a revised manuscript in Development. However as you will see the reviewer has raised a very minor point that I hope you can address in the very final version of your manuscript. If you do not agree with any of their criticisms or suggestions explain clearly why this is so. Your revised manuscript will not require any further review, rather I will look it over myself prior to acceptance.

© 2023. Published by The Company of Biologists under the terms of the Creative Commons Attribution License (https://creativecommons.org/licenses/by/4.0/).

Reviewer 2

Advance summary and potential significance to field

Sterile hosts are a valuable tool for the generation of novel chicken lines etc. This paper represents an alternative drug-inducible system to the current approaches available. Additionally, the system presented here results in non-transplanted red fluorescent primordial germs cells from the first stages of development in the second generation of birds.

Comments for the author

On the whole, all reviewers comments have been well addressed. The major concerns around inclusion of literature reporting similar approaches have now been addressed. The authors have also indicated clearly where the questions raised cannot be addressed within the timescale of a revision (namely the generational work).
Last, if minor point, while reviewer 1 comment 5 regarding numbers has been addressed, the way they are presented is confusing and could do with some clarifying e.g. '10/12; 6 gSAMURAI embryos' is not entirely clear in my opinion.

Second revision

Author response to reviewers' comments

Response to Reviewer's comment

I appreciate the reviewer for the valuable suggestion. I have now revised the manuscript by changing the text and Fig. S3.

Reviewer #2 comment.
Last, if minor point, while reviewer 1 comment 5 regarding numbers has been addressed, the way they are presented is confusing and could do with some clarifying e.g. '10/12; 6 gSAMURAI embryos' is not entirely clear in my opinion.

>> I changed the sentence (lines 223-226, shown in red), and modified Table S3 to show the number of surviving embryos at E10 in gSAMURAI and control cases, respectively. We hope this will avoid being confusing and misleading.

Third decision letter

MS ID#: DEVELOP/2023/202079

MS TITLE: An inducible germ cell ablation chicken model for high-grade germline chimeras

AUTHORS: Yi-Chen Chen, Daisuke Saito, Takayuki Suzuki, and Tatsuya Takemoto
ARTICLE TYPE: Techniques and Resources Article

I am happy to tell you that your manuscript has been accepted for publication in Development, pending our standard ethics checks.

© 2023. Published by The Company of Biologists under the terms of the Creative Commons Attribution License (https://creativecommons.org/licenses/by/4.0/).
